# Case-Based Immigrant Health Ethics Curriculum: A Pathway to Improve Care and Advocacy

**DOI:** 10.7759/cureus.49900

**Published:** 2023-12-04

**Authors:** Cara E Texler, Ashley K Fernandes, Abha H Athale, Carmen E Cobb, Stephanie M Lauden

**Affiliations:** 1 Hospital Medicine, Ohio State University, Nationwide Children's Hospital, Columbus, USA; 2 Pediatrics, Ohio State University, Nationwide Children's Hospital, Columbus, USA; 3 Pediatric Emergency Medicine, Ohio State University, Nationwide Children's Hospital, Columbus, USA; 4 Hospital Medicine, University of California San Francisco School of Medicine, San Francisco, USA; 5 Hospital Medicine, University of Colorado School of Medicine, Aurora, USA

**Keywords:** health advocacy, immigrant health, medical education, curriculum, cultural competency, clinical ethics

## Abstract

Introduction

Immigrants comprised a significant portion of the total population in the United States (US), and a considerable number of children in the US live with at least one immigrant parent, which has continued to increase over the past decades. However, healthcare providers (HPs) in the US report lack of comfort in interacting with immigrant and refugee populations.

Methods

The authors, in partnership with the Midwest Consortium of Global Health Educators, developed an innovative, interactive ethics curriculum within the Immigrant Partnership Advocacy and Curricular Kit (I-PACK). They sought to increase HPs' confidence in navigating complex encounters with immigrant families by teaching a relevant ethical framework, highlighting the importance of cultural humility, and equipping learners with an ethics tool (five-box Method) for use in clinical encounters. They piloted the curriculum during three workshop sessions in 2020-2021, and this curriculum continues to be used nationally as a part of I-PACK.

Results

Pre- and post-session surveys indicated that all participants (100%, n=22) reported acquisition of new skills/knowledge and 19 (86%) felt confident applying this to their clinical practice. The participants reported appreciation for an ethical framework with which to analyze cases, enjoyment of active participation in small group discussions, and utility of the five-box method tool. Some areas of improvement offered were to have more cases and more time dedicated to small-group discussions.

Conclusions

Given the success of the I-PACK ethics curriculum pilot, the authors plan to incorporate immigrant health cases in the general ethics training in medical school classes and pediatric residency training. Furthermore, they will advocate for the importance of including immigrant health ethics across graduate medical education, as fluency and competence in navigating the ethics of immigrant health are required to provide patient-centered, culturally informed care to all populations.

## Introduction

In the United States, in 2019, 26% of children lived with at least one immigrant parent, and immigrants comprised 13.7% of the total population in the United States (US) [1]. From 2016 to 2021, US refugee arrivals decreased due to political changes and the global pandemic; however, 2022 showed an increase in refugee admissions. Projections for 2023 indicate a significant increase in refugee arrivals, a trend which is expected to continue [2]. Global migrations due to conflict in Afghanistan, Ukraine, and Central America will continue to fuel the need for capacity to accept additional forcibly-displaced families. Moreover, immigrant and refugee patients access all levels of healthcare, including inpatient, outpatient, and emergency settings. Healthcare professionals (HPs) will continue to have increased interactions with members of these populations given expected increases in migration and known patterns of healthcare utilization [3-5].

While immigrant and refugee populations have distinct paths of migration, they share unique health care needs, along with language and cultural differences, which put them at risk for adverse events, miscommunication, and health disparities [3,6-11]. Given that our ethics curriculum focuses on cultural humility and healthcare interactions across discordant languages and cultures, for simplicity, when referring to “immigrant patients/families,” we also include refugee patients/families in this paper, focusing specifically on how to address the education of trainees for both populations.

Prior research has shown that US HPs report discomfort interacting with immigrant populations [12-14]. Differences in language, cultural values, and beliefs about disease influence how individuals participate in and experience the healthcare system [3,6-11]. When Western-trained HPs approach immigrant families through their own cultural lenses, misunderstandings and ethical dilemmas may arise, threatening the development of fruitful HP-family relationships, which affect patient care.

Gaining fluency in ethical challenges experienced by immigrant families is critical for HPs. Within the authors’ field of pediatrics, the Accreditation Council for Graduate Medical Education and the American Board of Pediatrics recognize the value of providing instruction in cultural humility, ethics, and professionalism to trainees, yet there is a surprising dearth of graduate medical education (GME) at the nexus of immigrant health and bioethics [15-16].

Increased interactions between HPs with immigrant families coupled with HPs feelings of being unequipped to care for these specialized populations highlight the importance of integrating immigrant health in GME and training. To address this need, the Midwest Consortium of Global Child Health Educators (MCGCHE) developed the Immigrant Partnership and Advocacy Curricular Kit (I-PACK), a novel curriculum that addresses eight domains of education related to immigrant health. The development of the larger curricular package is described elsewhere [17]. Here, we describe the development, implementation, and assessment of the ethics portion of the curriculum. The full curriculum is available for free download at https://sugarprep.org/i-pack.

This article was previously presented as an oral presentation at the Association of Pediatric Program Directors Meeting on March 26, 2021.

## Materials and methods

The overarching goal of the I-PACK ethics curriculum is to equip HPs with an ethical framework to interpret and respond to ethical dilemmas when interacting clinically with immigrant patients/families. The pedagogic goal is to create a curriculum that is highly interactive, flexible in both required time and delivery setting, and inspires curiosity and confidence in working with immigrant families.

Curriculum development**

A team of experts in ethics, medical education, and immigrant health created the content for this curriculum using Kern’s six-step approach to curricular development [18]. The curriculum contains the following components: 1) ethics foundation and framework, 2) novel five-box method tool, 3) clinical case application, and 4) ethics primer. Editors of the I-PACK toolkit and members of the MCGCHE peer-reviewed the content.

Ethics foundation and framework

**Figure 1:**
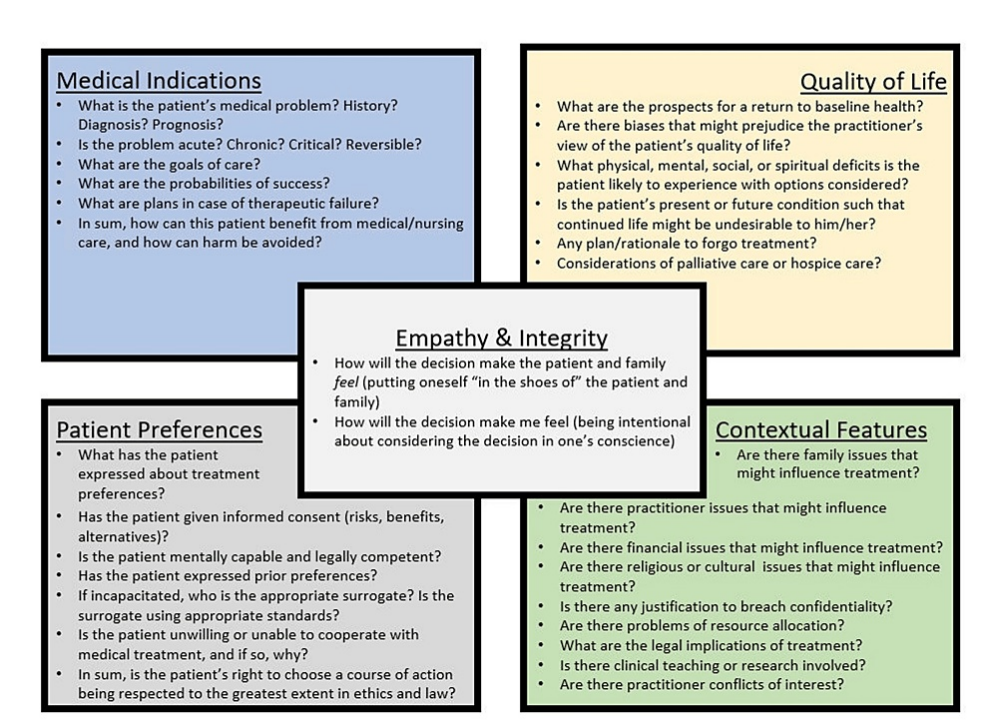
Five-box method tool for analyzing bioethical issues Adapted by A Fernandes from Jonsen et al. [19]. Fernandes received permission from the authors to create this adaptation.

The didactic component introduces cultural humility as it relates to ethics and equips participants with a tangible tool for analyzing ethics cases. This tool, the “five-box method,” is an adaptation by co-author Dr. Ashley Fernandes of Jonsen et al.’s four-box method tool [19]. Fernandes received permission from the authors to create this adaptation. The five-box method guides participants to analyze clinical ethical scenarios using five domains (Figure 1).

By featuring “empathy and integrity” as the new “fifth box” to Jonsen et al.’s existing method, HPs acknowledge how their emotions and morals influence interactions with their patients and team. The five-box method, which has been utilized successfully as a part of team-based learning exercises, provides shared structure to guide potentially difficult conversations among members of multidisciplinary healthcare teams. More details on the five-box method can be found here: https://bioethicstbl.org.

Clinical case application

After establishing a shared theoretical framework, learners may apply their knowledge to clinical cases. Content experts developed cases to represent immigrant families across many cultures and diagnoses. For example, cases highlight the challenges associated with utilization of medical interpreters, autonomy of adolescent patients, and child neglect. Case scenarios are designed to depict real-life complexities of caring for immigrant patients, while providing learners opportunities to practice ethical decision-making in a safe educational setting. For an example of the facilitator guide instructions and a case, please see the appendices. 

Primer

Anticipating desire for further reading, we developed an ethics primer as supplemental material. The primer includes expansion of ethical framework and theory, summary of ethics of cultural competence, and an example application of the five-box method tool.

Setting and facilitator tools

Before beginning conversations surrounding ethics, facilitators establish ground rules of respect, confidentiality, and trust. Facilitators should anticipate the need to redirect conversations if they become uncivil or intolerant.

The case-based component is designed for implementation in groups of five to 10 learners, where participants explore a case and discuss accompanying questions. We chose groups of this size to create intimacy in discussing sensitive topics. If delivering the curriculum in a larger group, participants are invited to share emotive and narrative responses to the cases in a large group reflection session, after small-group analytic discussions are complete.

A learner guide and facilitator guide accompany each case. The facilitator guide provides thought-provoking questions for case discussion, guidance regarding ethical framework application, and key learning points to deliver. In addition, references and resources related to each case are included for further facilitator preparation.

To respond to the dynamic clinical learning environments, this curriculum may be delivered either in-person or virtually. The curriculum is easily modifiable and may be delivered alone or combined with other I-PACK modules. In addition, the curriculum is designed to equip facilitators who may have minimal experience with ethical principles to facilitate meaningful dialogue regarding cases addressing the ethics of immigrant healthcare.

Curriculum implementation and evaluation

The author group implemented the ethics curriculum at two US pediatric academic centers, the Ohio State University and the University of Wisconsin, and one international conference, the North American Refugee Health Conference. Sessions were conducted virtually due to in-person meeting limitations associated with the global pandemic.

The participants completed pre-session and post-session surveys immediately before and after the workshop sessions. Both pre- and post-session surveys included 15 questions to assess knowledge, skills, and application of concepts presented. In addition, the pre-session survey asked demographic questions, and the post-session survey asked curriculum evaluation questions. All participant information was de-identified to preserve anonymity. This study was determined exempt from institutional review board approval by the Nationwide Children’s Hospital committee and guidelines due to not being considered research involving human subjects. 

Statistical analysis

Thirty-seven individuals completed the pre-session survey, and 22 of these individuals completed the post-session survey. As outcomes of interest were differences among individuals in the pre-session and post-session surveys, only those who completed both surveys were included in the analysis. Summary statistics for baseline demographics and outcomes of interest were calculated using descriptive statistics. All analyses were performed in R version 4.0 (R Foundation for Statistical Computing, Vienna, Austria).

## Results

Twenty-two participants completed both pre- and post-session surveys, and demographic information is summarized in Table 1.

**Table 1:** Demographic information of the study participants Statistics presented as n (%).

	n = 22
Healthcare provider	
Yes	18 (82)
No	4 (18)
Self-identifies as an immigrant	
Yes	3 (14)
No	19 (86)
Self-identifies as an adult child of an immigrant	
Yes	5 (23)
No	17 (77)
Frequency encountering immigrant or refugee families	
Daily	3 (14)
Weekly	11(50)
Monthly	7 (32)
Never	1 (4)
Percentage of patients seen who are immigrants or refugees	
< 20%	12 (55)
20-40%	7 (32)
41-60%	1 (4)
> 60%	2 (9)
Months working in healthcare in a low- or middle-income country	
> 2 months	7(22)
≤ 2 months	15(78)

The majority of participants (82%, 18/22) were HPs and 64% (14/22) reported encountering immigrant or refugee families at least weekly. In addition, 14% (3/22) self-identified as immigrants, while 23% (5/22) self-identified as adult children of immigrants.

**Figure 2:**
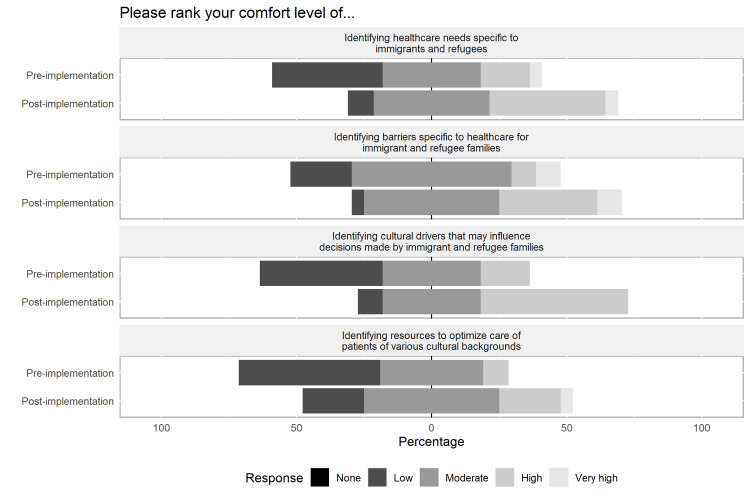
Pre- and post-session scores to assess concepts presented - comfort

**Figure 3:**
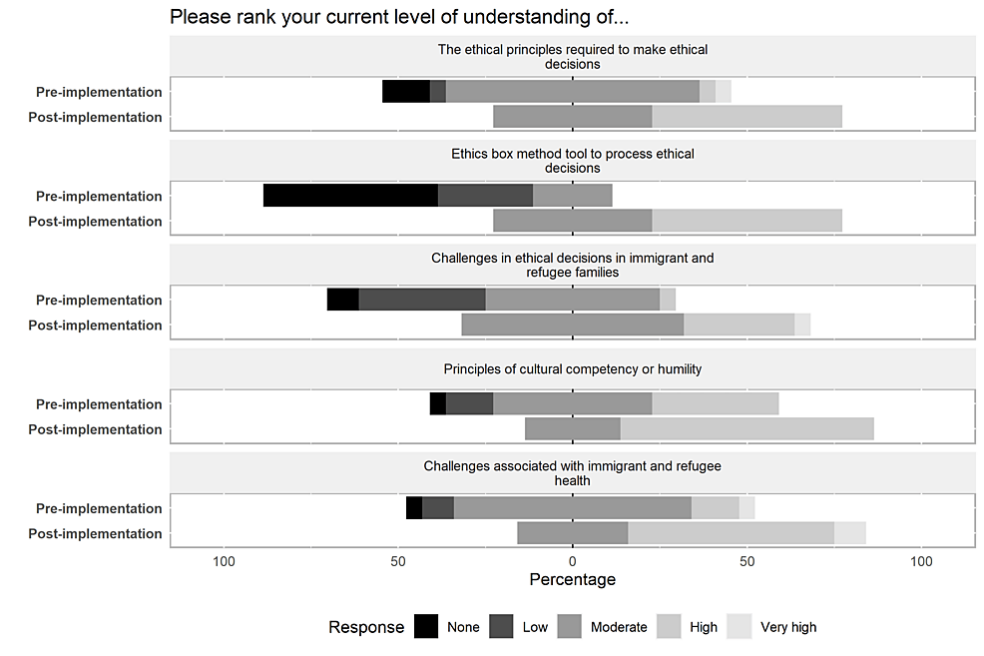
Pre- and post-session scores to assess concepts presented - understanding

**Figure 4:**
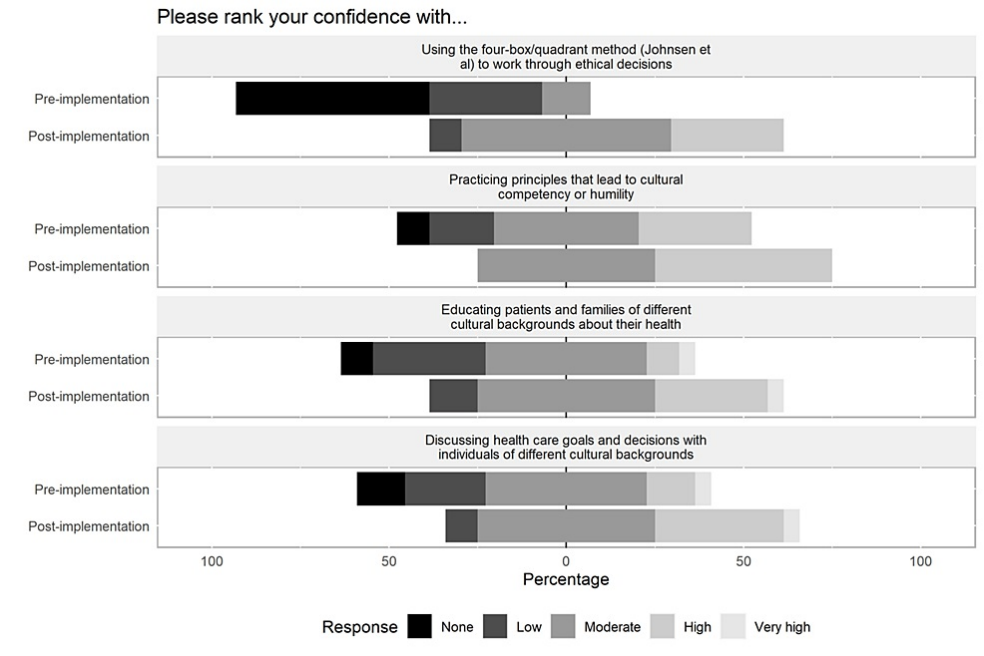
Pre- and post-session scores to assess concepts presented - confidence

There were no significant differences in baseline comfort and understanding when separated by reported baseline characteristics. After completing the session, all participants (100%, 22/22) reported acquisition of new skills/knowledge, and 86% (19/22) felt confident applying this to their practice. Results regarding changes in understanding, comfort, and confidence in managing ethical dilemmas regarding immigrant patients are summarized in Figures 2-4.

The comments, featured in Table 2, include successful aspects of the training and opportunities for improvement.

**Table 2:** Selected participants’ comments from the pre- and post-surveys

Participants’ comments regarding specific areas of success	Participants’ comments regarding specific areas for improvement
Framework for approaching cases. Increased my awareness of potential dilemmas that pass my clinical situations.	More group time rather than the time to spend sharing amongst groups - good to share more perspective but always feels surface level without the time to discuss.
Allowed for discussion that could be universally used in different fields of practice. It showed that collaboration on a particular case is needed and can be beneficial to have multiple viewpoints.	I would have liked to have time to go through more of the cases.
Combining a review of theory with a very realistic case. Use of a concrete thinking model (five-box) that I can easily imagine using to frame my in-the-moment thinking.	

## Discussion

In this report, we describe an innovative approach to incorporating immigrant health ethics training into GME, thereby filling a crucial curricular gap. Before receiving this training, the participants reported lack of knowledge and confidence in addressing clinical situations related to immigrant healthcare, reflecting established literature findings [12-14,20]. However, after participation, the respondents reported increases in understanding, confidence, and comfort regarding these encounters.

Similar to Mobasher's findings that medical ethics education can be successfully delivered virtually, our findings demonstrate that ethics training regarding immigrant health can be facilitated virtually [21]. Outcomes showed that delivery of workshops involving facilitated, small group discussions of even sensitive topics can be implemented successfully in a virtual format, which has potential to increase dissemination among institutions. We attribute the success to the following key factors: 1) facilitators created a culture of safety by encouraging confidentiality and judgment-free communication; 2) small-group size was limited to less than 10 participants, allowing all participants to see each other on one screen; and 3) participants were HPs who adapted quickly to a virtual learning environment format. Given our evolving learning environment, future ethics curricula should be designed to readily adapt to both virtual and in-person contexts.

In an era of competing medical education priorities, we also demonstrated that 20-minute case discussions were robust enough to stimulate substantive conversation, confidence, and learning regarding immigrant health ethics. Moreover, we found that learners find the use of a practical tool, the five-box method, helpful in navigating ethical dilemmas.

Anecdotally, resident physicians express feelings of helplessness, sadness, and frustration when faced with difficult clinical cases regarding immigrant children, particularly when the lack of eligibility for Medicaid or other health insurance programs limit medical treatment and management options. The five-box method’s acknowledgement of the emotions and conscience of the HP gives voice to this moral distress while providing structure needed to analyze patient encounters in both academic and pragmatic discussions. Given its utility, we continue to employ the five-box method Tool in ethics training.

By collaborating with multiple institutions via the MCGCHE and I-PACK, this ethics curriculum is one essential component of a comprehensive immigrant health curriculum. The partnership allows I-PACK modules to achieve increased dissemination while promoting teamwork across institutions. Furthermore, the MCGCHE commits to providing content freely available for download, thus increasing dissemination to individuals, institutions, and international partners. As our team develops new curricula, we will look to multi-institution networks for collaboration and dissemination.

Despite delivery in multiple settings with diverse groups of learners, our curricular implementation may have been limited by selection bias as many participants expressed baseline interest in either global health or immigrant health. Similarly, facilitators of the pilot implementation have expertise in ethics and/or global health, which may have impacted the delivery of the training. Other limitations include modest sample size and dropout rate of participants who did not complete both the pre- and post-surveys.

Since the pilot curriculum implementation, components of the I-PACK ethics curriculum have been incorporated into the core ethics curriculum at our institution with resident physicians. The curriculum is particularly well suited to resident physicians given their developing autonomy leading clinical discussions with multidisciplinary teams and families, active role in making clinical decisions, and ethical dilemmas they face as trainees. In contrast to more experienced attending physicians who express higher levels of comfort caring for immigrant children [22], resident physicians demonstrate discomfort, lower self-efficacy, and desire for more training in this area [12-14,20,23].

## Conclusions

The need to include immigrant health curricula into GME is achieving increased recognition. Gaining fluency and confidence in navigating the ethics of immigrant healthcare represents a key component of providing patient-centered, culturally informed care to all patient populations. Learners require repeated exposure to this content and ongoing opportunities to apply ethical analysis tools to their clinical encounters. Our curriculum provides a freely available, highly adaptable, and effective tool to meet this need, with no specific ethical expertise required. In future research, we recommend including family advisors who represent cultures featured in clinical cases to review curricular components. Furthermore, we recommend studies to evaluate the impact of such curricula on the patient experience, quality, and safety of healthcare received by immigrant families with whom we partner.

## Figures and Tables

**Figure 5:**
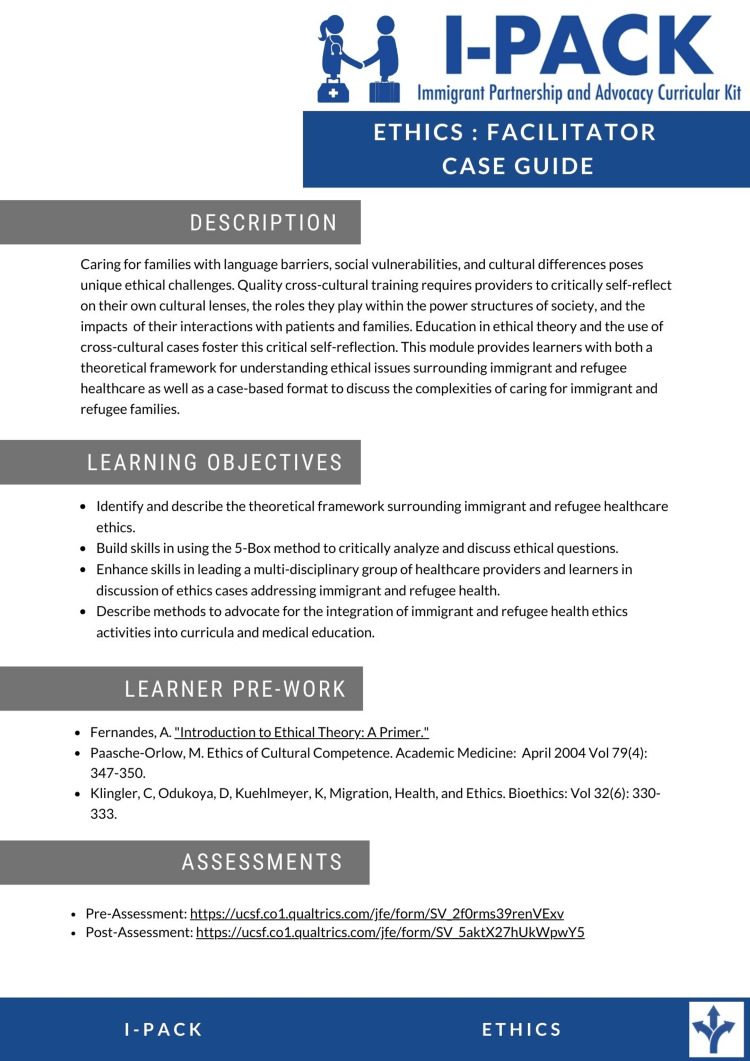
I-PACK ethics module facilitator guide instructions Materials are available for free download at https://sugarprep.org/i-pack/.

**Figure 6:**
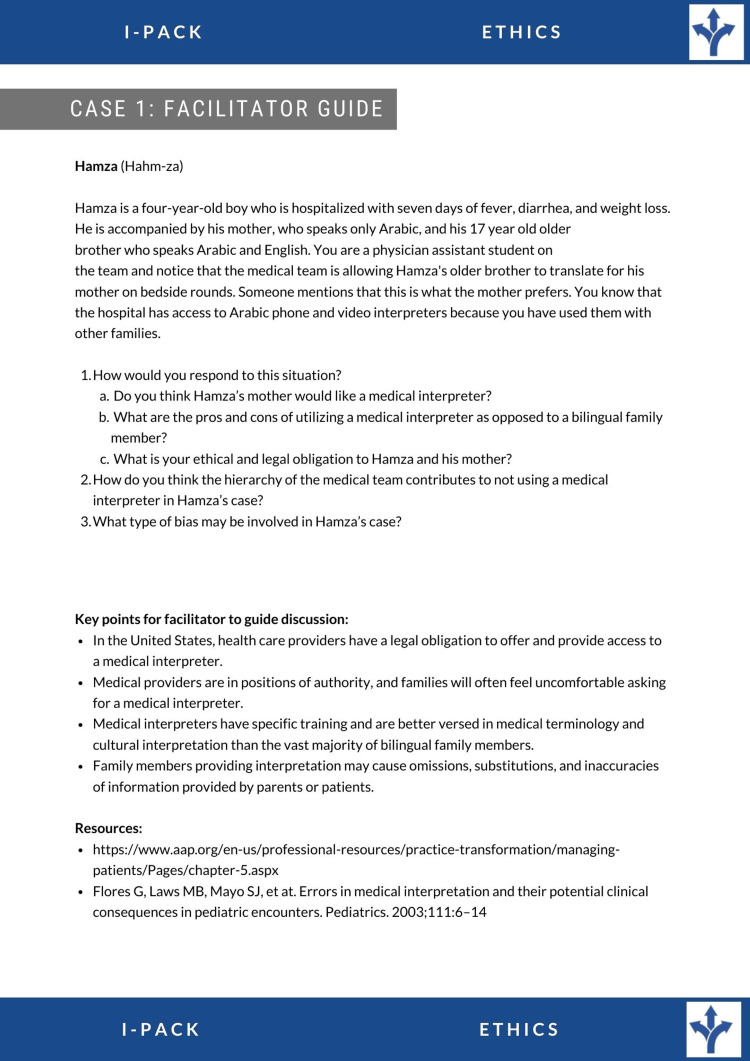
I-PACK ethics module facilitator guide case 1 Materials are available for free download at https://sugarprep.org/i-pack/.

**Figure 7:**
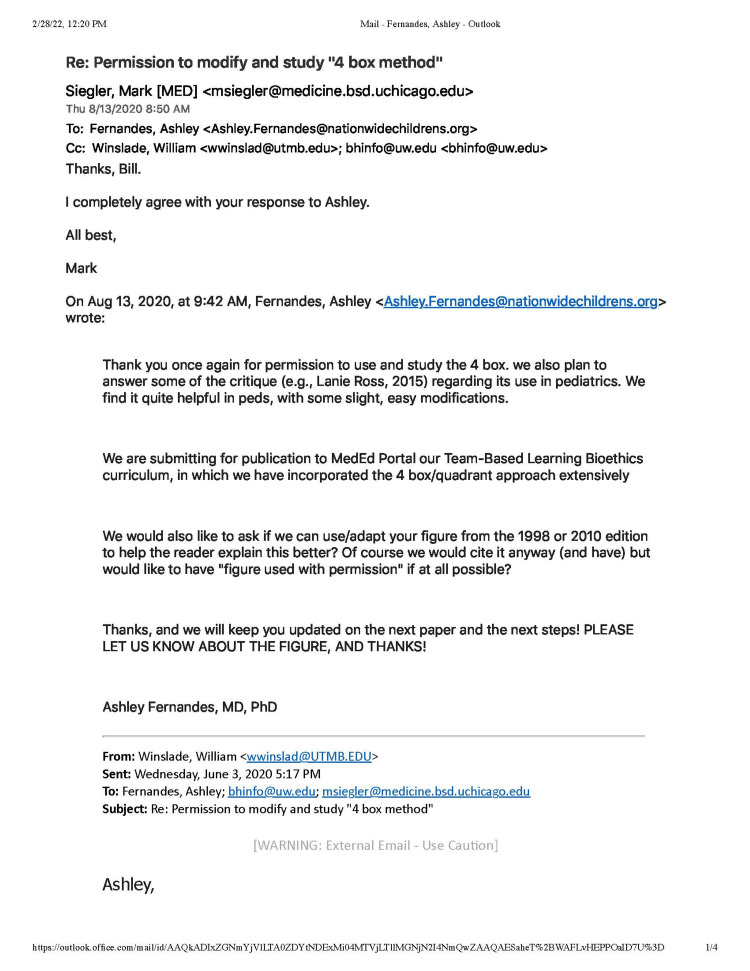
Permission for use of the four-box method tool by Jonsen et al.

**Figure 8:**
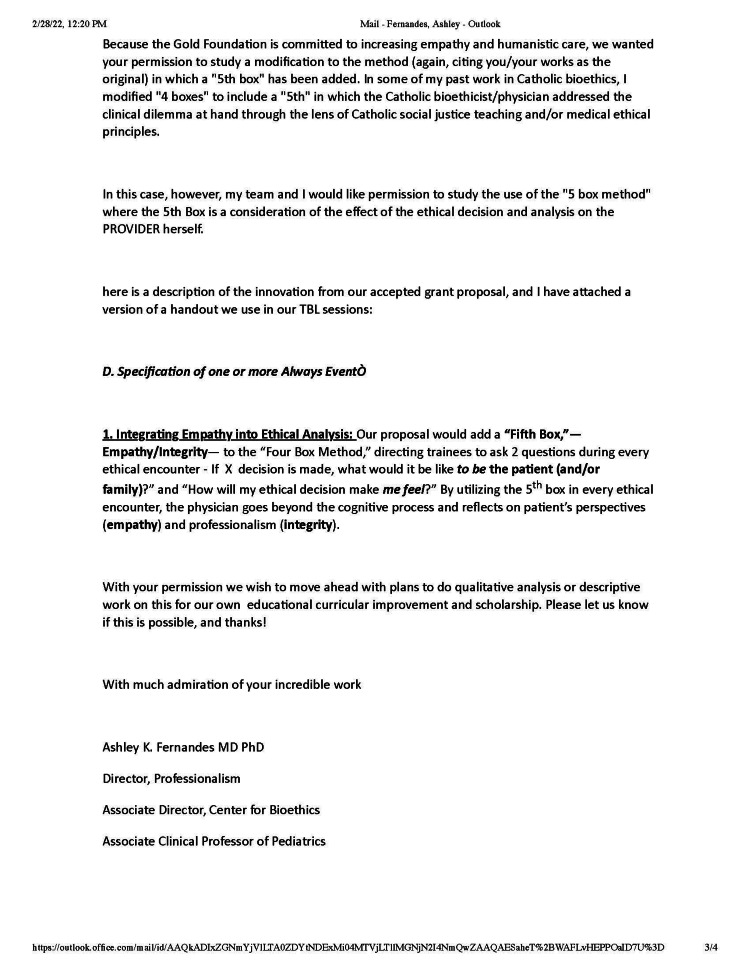
Permission for use of the four-box method tool by Jonsen et al.

**Figure 9:**
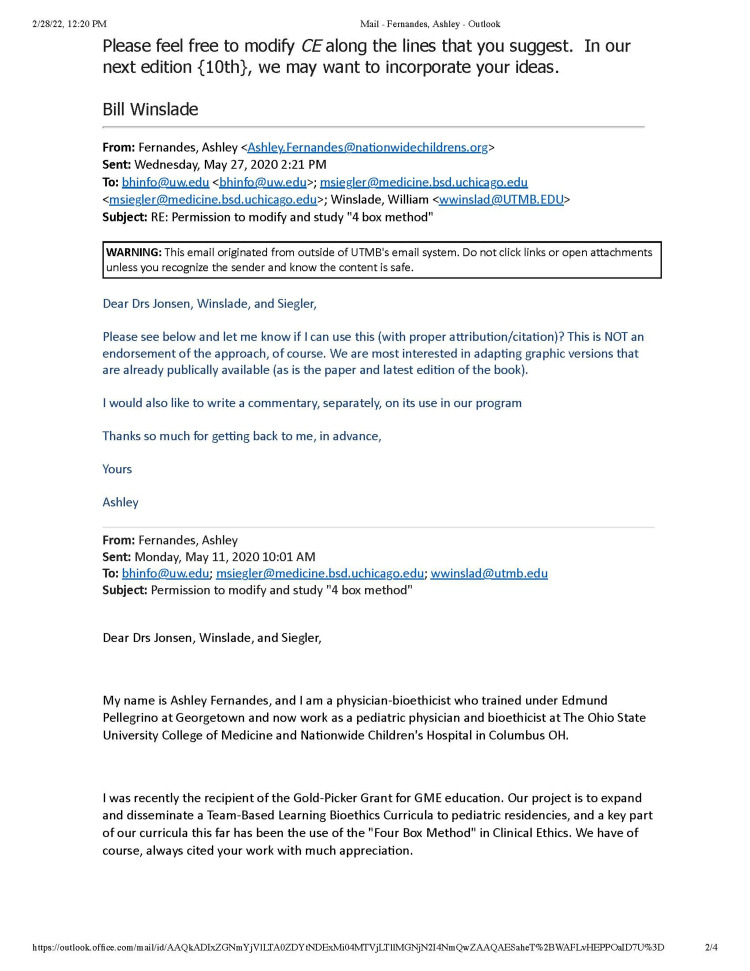
Permission for use of the four-box method tool by Jonsen et al.
